# Visceral Leishmaniasis Masquerading as Drug-Induced Pancytopenia in Lung Cancer Patients

**DOI:** 10.3390/curroncol31040168

**Published:** 2024-04-17

**Authors:** Sophie Laroumagne, Julie Tronchetti, Hervé Dutau, Philippe Astoul

**Affiliations:** 1 Department of Thoracic Oncology, Pleural Diseases, and Interventional Pulmonology, Hôpital Nord, Aix-Marseille University, 13015 Marseille, France; s_laroumagne@yahoo.fr (S.L.); julie.tronchetti@ap-hm.fr (J.T.); herve.dutau@ap-hm.fr (H.D.); 2 Faculty La Timone, Aix-Marseille University, 13005 Marseille, France

**Keywords:** lung cancer, chemotherapy, pancytopenia, visceral leishmaniasis

## Abstract

Maintenance chemotherapy is a standard treatment in patients with non-progressive advance staged IV non-squamous non-small cell lung cancer after induction therapy. Here, we report the case of a 53-year-old man undergoing a maintenance monotherapy with pemetrexed who presented prolonged pancytopenia despite filgrastim injections. A bone marrow aspiration revealed a macrophage activation syndrome with *Leishmania* amastigotes. A Polymerase Chest Reaction testing confirmed the diagnosis of visceral leishmaniasis. Treatment with liposomal amphotericin B was started. Oncologists should bear in mind that visceral leishmaniasis in endemic areas can potentially induce severe and prolonged pancytopenia in immunosuppressed patients, during chemotherapy in particular.

## 1. Introduction

Maintenance chemotherapy is a standard treatment in patients with non-progressive advanced non-squamous non-small cell lung cancer after induction therapy. If this therapeutic regimen has a low incidence for bone marrow toxicity, pancytopenia can occur, leading to delays for further cycles precluding an optimal management [1]. Nevertheless, other causes of anemia, neutropenia, and thrombocytopenia can be found in immunocompromised patients. Among them, infections with a protozoan parasite of genus leishmania acquired by the bite of a sandfly is increasingly regularly in patients with immunosuppression related to human immunodeficiency virus (HIV), immunosuppressive treatments, organ transplantations, and neoplastic diseases [2]. Here, we report the case of a lung cancer patient undergoing maintenance chemotherapy, who presented prolonged pancytopenia, presumably drug-induced, but in fact related to a visceral leishmaniasis (kala azar).

## 2. Case Report

A 53-year-old man, a resident of a Mediterranean coast area (south of France), was admitted with a history of fever, fatigue, no weight loss, and prolonged pancytopenia beginning three weeks after a first cycle of maintenance chemotherapy with pemetrexed (500 mg/m^2^) after 4 cycles of chemotherapy combining cis-platinum and pemetrexed, an antimetabolite drug that interferes with enzymes involved in DNA synthesis and also the folate-dependent metabolic processes necessary for DNA replication and homocysteine homeostasis. Except for his neoplastic disease (extended stage 4 lung adenocarcinoma—pT1bN2M+—Non mutated EGFR—ALK, ROS, PDL1 negative), there was no past medical history, in particular, no blood transfusion nor travel abroad. His ECOG performance status was 1, Body Mass Index (BMI) 27, and Body Surface Area (BSA) 2. The physical examination revealed pallor and moderate splenomegaly without peripheral lymphadenopathy. No skin lesions were apparent.

**Figure 1:**
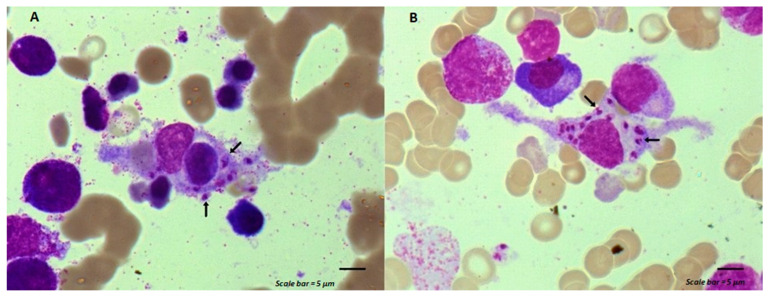
Macrophage activation syndrome associated with *Leishmania* infection. (**A**) *Leishmania* amastigotes (arrows) associated to a macrophage that has engulfed a lymphocyte. (**B**) *Leishmania* amastigotes (arrows) within the cytoplasm of a macrophage. Scale bar = 5 µm.

Despite filgrastim injections (subcutaneous daily injection of 5 µg/kg for 10 days), laboratory studies showed a white-cell count of 1600/mm^3^ (range, 4500 to 10,000), a neutrophile count of 570/mm^3^ (range, 2000 to 5000), a eosinophil count of 0/mm^3^ (range, 0.10 to 0.500), a platelet count of 70,000/mm^3^ (range, 150,000 to 400,000), and a hemoglobin level of 7.7 g/dL (range, 13.0 to 16.0), MCV 83 fl (range, 82 to 98), and MCH 26.1 pg (range, 27 to 32). His C reactive protein (CRP) test was 105.3 mg/L (range, 0.0–5.0). Renal and liver function tests, blood glucose, electrolytes, serum ferritin, and iron were normal. Plasma levels for vitamin B12 and folate were normal, with 253 pmole/(range, 145 to 569) and 27.1 nmole/L (range, 8.83 to 60.8), respectively. Blood cultures and tests for severe acute respiratory syndrome coronavirus 2 (SARS-CoV-2) and human immunodeficiency virus (HIV) were negative, and cytomegalovirus infection was ruled out. Bone marrow aspiration cytology revealed a normal trilineage hematopoiesis with *Leishmania* amastigotes out (Figure 1A, arrow) and within cytoplasm of a macrophage (Figure 1B, arrow). Polymerase chain reaction (PCR) testing on stained slides with May-Grunwald Giemsa (MGG) confirmed the diagnosis of visceral leishmaniasis. The maintenance chemotherapy was suspended. A treatment with liposomal amphotericin B (L-AmB) was started, targeting a total dose of 40 mg/kg (4 mg·kg/day from day 1 to day 5 and weekly till day 38) with weekly blood PCR testing [3]. Negativation of blood PCR for leishmania occurred 4 weeks after the beginning of the dedicated treatment.

## 3. Discussion

Leishmaniasis refers to various clinical syndromes caused by infection with a protozoan parasite acquired by the bite of phlebotomine females, common name sandfly. The last are hematophagous flies which transmit *Leishmania infantum* and spread leishmaniasis. It is endemic in the Mediterranean area and dogs provide the reservoir [4]. Usually, the common presentation combines fever, chills, weight loss, hepatosplenomegaly, and skin hyperpigmentation with pancytopenia on blood examination in infants and small children. However, in the last few decades, the medical situations leading to a patient’s immunosuppression (HIV infections, organ transplantations, and immunosuppressive treatments including prolonged steroid therapy and chemotherapy) dramatically increase the risk for leishmania-infected people to develop visceral disease. If the introduction of the highly active antiretroviral treatment (HAART) decreased the number of cases of visceral leishmaniasis in HIV patients, conversely, the disease is increasing in immunocompromised HIV negative patients. In these cases, atypical signs and low-grade fever can foster the risk of fatal diagnostic delay, poor response to therapy, and at the same time, preclude the optimal cancer therapeutic strategy.

Visceral leishmaniasis and solid cancer may be coincidental and different associations can be seen. Among them, simultaneous diagnosis of leishmaniasis and a neoplastic disorder in the same tissue samples of immunocompromised patients, leishmaniasis mimicking a malignant disorder (e.g., lymphoma), direct involvement of Leishmania in the pathogenesis/occurrence of malignant lesions (e.g., skin), and leishmaniasis in patients receiving chemotherapy for various malignant disorders are difficult to diagnose and treat [2]. Visceral leishmaniasis can happen in these cases after a prolonged regimen of chemotherapy leading to a long period of immunosuppression. Our patient was treated by maintenance chemotherapy with pemetrexed for stage IV non-epithelial non-small cell lung cancer, which usually has a low incidence of bone marrow suppression (less than 5%) [5]. To our knowledge, it is the first case of leishmaniasis-malignancy association during a maintenance therapy. In accordance with the literature, we used L-AmB as induction therapy because of its safety profile and good efficacy, including for immunocompromised patients (targeted total cumulative dose of 40 mg/kg body weight) [6]. Chemotherapy with pemetrexed was interrupted during the initiation of antileishmanial therapy with L-AmB but then resumed because of cancer progression assessed by a chest computed tomography (CT) scan two months later. Reintroduction of pemetrexed during leishmaniasis therapy was uneventful.

Leishmaniasis can directly or indirectly affect the presentation, diagnosis, and course of malignant disease, especially in patients undergoing chemotherapy. This should be considered in the differential diagnosis of malignancies in geographic areas, in particular, Southern Europe along the Mediterranean basin, where it is endemic, and/or in patients with travel history to these areas [7,8]. Oncologists should bear in mind that visceral leishmaniasis can potentially induce severe and prolonged pancytopenia in immunosuppressed patients, in particular, during chemotherapy in an endemic area.

## Data Availability

The data presented in this study are available on request from the corresponding author. The data are not publicly available due to privacy and ethical reasons.
